# Social and Poor-Law Problems

**Published:** 1909-04-24

**Authors:** 


### SOCIAL AND POOR-LAW PROBLEMS.

### THE EDUCATION OF POOR-LAW CHILDREN.

The report of the educational work done in Poor-law schools by Mr. Tillard and Miss Synge, which was recently published, draws attention to many points which are not fully realised even by Boards of Guardians, and certainly not known to the general public. In those circumstances the State Children's Association has sent out a letter which brings forward some facts which are well worth attention.

So far as the buildings and the staff go, Poor-law schools compare favourably with many others. The premises are for the most part spacious and airy, and the proportion of certificated teachers employed is as large as that of well-staffed elementary schools. As the classes in the Poor-law schools are smaller than in ordinary schools, the pupils have the advantage of greater individual attention on the part of the teachers. Yet the children in Poor-law schools are, in the opinion of the inspectors of the Board of Education, about a year behind those in ordinary schools in point of attainments. This might—and in some cases must—be set down to a poor heredity and a bad early environment; but these disadvantages are by no means universal. Many children of the State come under the Poor-law only through the death of their fathers. Until their orphanhood they had the same conditions of life as other children of their class, and after they go to the Poor-law school the advantage is with them so far as physical conditions go. They are better fed, better clothed, and better lodged than the majority of their former companions. Moreover it is found that Poor-law children who attend public elementary schools do exceedingly well. Why do they not do equally well in the Poor-law schools?

The answer may be given in two words—monotony and isolation, which injuriously affect both teachers and pupils. The average child of the humbler classes has his school hours, but also his home life, with all its varied interests, its amusements, even its anxieties. The teachers in elementary schools can, when their daily work is over, choose their own environment. The children of the richer classes are often sent to boarding-schools, but they have long holidays, when other influences than those of the school and its discipline affect them. But Poor-law children spend years in the school, with only rare changes of environment, or, it may be none. And everything around them tends to diminish any individuality or initiative which they may possess. Where numbers are large discipline needs must be strict, and the discipline lasts for 24 hours of the day. Even play is too thoroughly "organised."

"Organised games," say the Inspectors, "are gaining favour, and in some schools the right spirit prevails, but in the hands of some of the teachers they were dreary indeed. The children went through them as joylessly as if they were lessons; they did not understand what they were doing, and instead of bringing out the natural merriment of healthy children, the games seemed to sadden and destroy spontaneous play."

The inspectors think that Poor-law girls in particular need physical training, and that they do not get it.

"Their lives are full of dull routine and monotony. Scrubbing, which occupies much of their time, is no substitute for physical training, and is certainly not exhilarating. A great deal of their time is spent sitting over needle-work out of school hours, and they have less free time and recreation than the boys."

And unfortunately even as an industrial training this scrubbing is not successful. Again, the Inspectors say: "For the most part we found that while all the girls did a certain amount of washing and ironing, their work was incomplete; that is to say, they would iron collars they had not learned to starch and wash articles they never learned to iron, exactly in the same way in which they peeled potatoes that they could not cook. They assisted as 'hands' in the work of a large laundry; but for that very reason they failed to acquire any practical knowledge of the complete art of washing clothes. In the large institutions the cooking is done wholesale, but such work in such kitchens is no training for the girls for situations in small households or for their own homes when they get married."

And we may add to the inspectors' remarks that by way of keeping them unfit for the all-round work of the wife and mother in a humble home many Guardians refuse to send the girls to situations where only one servant is kept. That is, they are prevented from learning their future work in those small middle-class households where perhaps the best training in all-round housekeeping and economy is to be found.

The Inspectors suggest that, in view of the inevitable limitations of life in a Poor-law school, the training should be made more practical. The children "lack ordinary knowledge of the world and the transactions of everyday life, they do not handle money, and do not get to know the prices of articles in common use." The misfortune of this is obvious, but it is not clear how it can be remedied under the conditions of institutional life. We believe that Guardians, officials, and teachers do their best for the children under their care, and there is a general and sincere desire to give the children a really good education and a proper preparation for life. But the defects which the inspectors deplore seem to be inseparable from large institutions. The question then arises: Are these institutions necessary? The State Children's Association is opposed to them, and we think with reason. In a great number of cases these children are the offspring of widows who cannot unaided maintain them, but with some help—the outdoor relief which is so freely granted to many who are undeserving—could keep their homes together and bring their children up on wholesome, homely, practical lines, while the means of education are everywhere within reach of all. If the children are wholly orphaned, but come of a respectable family, it should not be impossible to find a respectable home for them with people of their own class.

---

One of the special features of the Missionary Exhibition, entitled "Africa and the East," which will be held at the Royal Agricultural Hall from June 8 to July 3, under the auspices of the Church Missionary Society, will be a special exhibit of outfit suitable for missionaries and travellers shown in a special outfit section. This section is being arranged under the auspices of Livingstone College, which is specially devoted to the study of all questions affecting the health of missionaries. It is hoped on the present occasion to demonstrate the most up-to-date methods of modern outfitting for travellers in the tropics. One of the special features of this section will be an exhibition of the various methods of protection from mosquitoes and other insects. The organiser of this section, Dr. C. F. Harford, Principal of Livingstone College, will be pleased to communicate with any who desire particulars if they will write to him at Livingstone College, Leyton, E.

